# Unveiling the exodus of nurses in Somalia: exploring the reasons for nurse attrition and the implications for the healthcare system

**DOI:** 10.1186/s12912-026-05021-z

**Published:** 2026-07-14

**Authors:** Hassan Abdullahi Dahie

**Affiliations:** SOS College of Health Science University, SOS Children’s Villages-Mogadishu, Mogadishu, Somalia

**Keywords:** Former nurses, Healthcare administrators, Nurse attrition, Patient care

## Abstract

**Background:**

The indispensable role of nurses in healthcare systems is widely recognized, as they contribute significantly to the effectiveness and continuity of healthcare delivery. However, nurse attrition has emerged as a growing concern in fragile and resource-limited settings such as Somalia, with serious consequences for health service provision.

**Objective:**

This study aimed to explore the key factors contributing to nurse attrition in Somalia and to examine the perceived implications of nurse attrition for the healthcare system from the perspectives of former nurses and healthcare administrators.

**Methodology:**

A qualitative descriptive study was conducted in the Benadir region, involving 42 former qualified nurses and 8 healthcare administrators, representing both genders. In-depth interviews were conducted with former nurses to explore their motivations for entering the nursing profession, their professional experiences, and the factors leading to their attrition. Semi-structured interviews with healthcare administrators focused on understanding the broader implications of nurse attrition within the healthcare system. Data were analyzed using reflexive thematic analysis to identify and interpret central themes.

**Results:**

Nurses often enter the profession driven by compassion and the desire to impact patient lives positively. However, disparities between initial expectations and realities of nursing practice, including inadequate compensation, burnout, unfavorable working conditions, job dissatisfaction, strained nurse-doctor relationships, and community misconceptions about the nursing role, contribute to nurse attrition. These contributory factors have several negative consequences, including a shortage of nurses, reduced care quality, increased workload for remaining nurses, longer patient wait times, limited access to healthcare services, higher operational expenses, added responsibilities for doctors, and lasting effects on public health.

**Conclusion & recommendations:**

Nurse attrition is a complex process influenced by various factors. To mitigate its impact on healthcare systems, policymakers and healthcare administrators must allocate resources strategically, focus on comprehensive planning, and prioritize the recognition, support, and retention of nurses within the healthcare framework.

**Supplementary Information:**

The online version contains supplementary material available at https://doi.org/10.1186/s12912-026-05021-z.

## Background

The indispensable role of nurses as the cornerstone of the healthcare system is evident, as their multifaceted contributions are instrumental in ensuring the optimal functioning and effectiveness of the entire healthcare ecosystem [1]. In healthcare, nursing is frequently heralded as a cornerstone profession essential for ensuring quality patient care and promoting overall well-being. Globally, there is a growing recognition among the nursing profession, healthcare policymakers, and society as a whole to analyze both the substantial contribution of nursing to healthcare and its associated costs [2, 3].

The nursing profession is widely regarded as the backbone of the health system due to its role as the primary point of contact and continuity of care for patients throughout their healthcare journey. Nurses play a pivotal role in various aspects of healthcare delivery, encompassing essential functions such as patient advocacy, direct patient care, health education, care coordination, emotional support, health promotion, preventive care, and research and innovation [4–6]. Nurses are key players in health promotion and disease prevention and the backbone of health care systems worldwide, particularly in nations affected by crises. Nurses work on the front lines of disease prevention, health promotion, and health management and are often the unsung heroes in health care facilities and emergency response. Despite the critical role they play in health care, there is a nursing shortage across the world that will affect the delivery of competent nursing care [7, 8]. According to the World Health Statistics Report, there are approximately 29 million nurses and midwives globally [9]. For all countries to reach Sustainable Development Goal 3 on health and well-being, WHO estimates that the world will need an additional 9 million nurses and midwives by the year 2030 [10].

The shortage of healthcare professionals is particularly prevalent in East Africa, with Somalia being one of the countries significantly affected by this issue due to its weak and fragile health system [11]. In spite of the prevailing scarcity of nursing personnel within the nation, a substantial number of nurses were disengaging from the profession through various avenues including early retirement and attrition [12]. Nurse attrition refers to the departure of nursing staff from their positions within healthcare facilities. In view of the above, this study investigates the factors contributing to nurse attrition and the consequences it carries for healthcare systems.

## Methods

### Study design and setting

A qualitative descriptive research design was employed to explore the reasons for nurse attrition among former nurses in Somalia. This approach was selected because it supports a clear, practice-oriented summary of participants’ accounts and is well suited to studies that seek to describe factors, conditions, and perspectives in everyday language [13]. The study was conducted in Benadir Region, Somalia [14].

### Study population and sample

The study population consisted of 42 qualified nurses who were previously employed within the healthcare system of Somalia but subsequently chose to leave the profession and 8 current health administrators who are managing public and private hospitals in the country. These individuals were selected as the focal point of investigation to explore the underlying reasons for nurse attrition and illuminate the potential implications of this phenomenon on the broader healthcare system.

### Data collection tool

In this study, the researcher utilized interview guides that were specifically tailored for former nurses and healthcare managers. The qualitative semi-structured interview guide for this study was formulated using the framework developed by Kallio et al. [14]. The former nurses’ guide encompassed inquiries into their intrinsic and extrinsic motivations for pursuing a nursing career, an in-depth exploration of their roles and experiential insights, an examination of personal and professional factors contributing to attrition from the profession, an analysis of the challenges encountered during their tenure, and a comprehensive assessment of the rewards derived from their nursing practice. On the other hand, the interview guide intended for healthcare managers adopted a broader perspective, centering on the systemic implications of nurse attrition within healthcare systems. It sought insights into strategies aimed at mitigating nurse attrition, bolstering nurse retention, and enhancing overall job satisfaction within the nursing profession (Supplementary file 1). The interview guides were structured to span a duration of 30 to 45 min.

### Participant selection

#### Inclusion and exclusion criteria

Former nurses were eligible if they held a recognized nursing qualification, had practiced nursing in Somalia for at least 12 months, and had exited nursing practice at the time of interview. Individuals were excluded if they had never practiced nursing (e.g., students), were currently employed as nurses, or were unable to provide informed consent. Healthcare administrators were eligible if they held managerial roles with responsibility for staffing and service delivery.

#### Recruitment

Participants were identified through professional networks and health facility contacts and were approached directly. Interested individuals received study information, and verbal informed consent was obtained prior to interview scheduling. The verbal consent procedure was reviewed and approved by the institutional ethics review board. Recruitment continued until data saturation was reached, with no new substantive insights emerging.

#### Data collection procedure

Interviews were conducted in a comfortable and private setting to foster an atmosphere conducive to open and authentic discussions. Participants were briefed on the study’s objectives, confidentiality measures, and their rights as research subjects. With participants’ consent, interviews were audio-recorded to guarantee accurate documentation of responses, allowing researchers to focus on engaging with participants during the discussions. The primary author of the study, who had no supervisory authority over participants, conducted the interviews with support from research assistants proficient in the local languages spoken by the interviewees. To minimize researcher bias, the interviewer maintained reflexive notes during data collection and analysis to document assumptions, emerging impressions, and potential influences on interpretation. The research team also discussed emerging codes and interpretations to ensure that findings remained grounded in participants’ accounts rather than the researchers’ prior expectations. This approach was taken to facilitate effective communication and comprehension during the interviews, ensuring that valuable data was collected in a linguistically and culturally sensitive manner.

#### Data management

Upon completion of the interviews, the audio recordings were transcribed verbatim to ensure accurate representation of the participants’ narratives. These transcriptions were systematically organized and stored in secure digital repositories. Rigorous measures were undertaken to maintain confidentiality, with the removal of any identifying information to safeguard participant anonymity.

#### Data analysis (Reflexive thematic analysis)

Reflexive thematic analysis was used to analyze the interview data and to identify recurring themes, patterns, and variations across participants’ accounts. The analysis followed several iterative stages, including familiarization with the data, initial coding, generation of candidate themes, review and refinement of themes, and final definition and labeling of themes [15]. To prevent premature closure of analysis, data were revisited across multiple analytic cycles, with earlier transcripts re-examined after later interviews. Analytic memos were maintained, and attention was given to identifying divergent or contradictory accounts before finalizing themes. In line with recommended mitigation strategies, themes were finalized only when they moved beyond simple topic summaries and demonstrated internal coherence, clear distinction from other themes, and sufficient evidential support across the dataset [16–19].

### Trustworthiness

Trustworthiness was enhanced through several strategies. Credibility was supported by using semi-structured interview guides, conducting interviews in private settings, audio-recording interviews with participants’ consent, and transcribing interviews verbatim. Dependability was strengthened through the use of a consistent data collection process across participants and iterative review of transcripts during analysis. Confirmability was supported by maintaining analytic memos, revisiting earlier transcripts during later stages of analysis, and grounding themes in direct participant quotations. Transferability was supported by providing contextual information on the study setting, participant groups, and the Somali healthcare system.

#### Ethics approval and consent to participate

The study obtained ethical approval through a comprehensive review process conducted by the institutional ethics review board of SOS College of Health Science University (Ref: 2023/SOS-IRB/CNS/P003). The study was conducted in accordance with the ethical principles of the Declaration of Helsinki, including respect for participants, voluntary participation, informed consent, confidentiality, privacy, and protection of vulnerable groups. This oversight body evaluated the study’s adherence to ethical guidelines, ensuring participant welfare, informed consent, confidentiality, and protection of vulnerable groups. Regarding participant’s consent, all prospective participants in this study were provided with a clear and detailed explanation of the research, and their informed consent was obtained verbally by the principal researcher. The verbal informed consent procedure employed in this study was duly reviewed and approved by the ethics committee of SOS College of Health Science University prior to the commencement of the research. This ensured that participants willingly participated in the study, with the assurance of privacy, confidentiality, and the freedom to withdraw without any repercussions.

## Results

### Demographic characteristics

A total of 50 participants (42 former nurses and 8 healthcare managers) participated in the study. The demographic profile of former nurses who transitioned out of the profession revealed key trends. The majority were male (73.8%) and belonged to the over-35 age group (50.0%). Most held bachelor’s degrees (78.6%) in terms of education. Additionally, a significant majority were married (59.5%) and had completed their studies in Somalia (90.5%), with a smaller percentage pursuing education abroad (9.5%). These findings provided valuable insights into the characteristics of former nurses who had moved on from their nursing careers. Conversely, concerning healthcare managers, the predominant group consisted of males (75%) who were aged 35 years or older. It’s worth noting that all the interviewed healthcare managers were married, and half of them (50%) had achieved a master’s degree obtained primarily from international universities (Table 1).

**Table 1:** Summary of themes and subthemes

Main Theme	Subthemes
Initial expectations of the nursing profession	Compassion and desire to help patients; expectation of fair remuneration; expectation of social recognition; desire for professional growth and flexible work
Experiences during nursing practice	Direct patient care roles; specialized and educational roles; rewarding patient interactions; limited involvement in research, policy, and administration
Discrepancy between expectations and reality	Heavy workload; limited income; low professional recognition; limited career guidance; misunderstanding of the nursing role
Reasons for leaving the nursing profession	Inadequate compensation; burnout and stress; unfavorable working conditions; job dissatisfaction; strained nurse-doctor relationships; community misconceptions about nurses
Implications of nurse attrition for the health system	Shortage of nursing staff; increased workload for remaining nurses; longer patient waiting time; reduced quality of care; reduced access to services; increased operational costs; additional burden on physicians; long-term public health effects

### Former nurses’ initial expectations regarding nursing profession

Participants shared their diverse motivations for initially pursuing a career in nursing, with common themes emerging from their responses. Many participants expressed a deep sense of compassion and a strong desire to make a positive impact on people’s/patient lives during times of vulnerability. They cited the prospect of playing a crucial role in patient care and contributing to their well-being as significant driving forces. In addition to their initial enthusiasm and dedication for the profession, the participants’ anticipations regarding a nursing career encompass equitable remuneration, societal recognition, moral fulfilment, flexibility in job roles, chances for professional growth, and leisure time.

When I started nursing, my primary goal was to find a profession I’m passionate about, one that offered a good salary, and earned respect in the community. That’s what I aspired to. (Participant 3, former nurse, male, left nursing 5 years ago).

For me, nursing was about having a flexible job, opportunities for growth, and earning well. I wanted a career that aligned with my interests and provided financial stability. (Participant 1, former nurse, male, left nursing 15 years ago).

### Nurses’ experience during nursing profession

Former nurses recounted memorable experiences in patient care and education. They brought a wealth of diverse experiences to the forefront of their nursing careers, showcasing the multifaceted nature of the profession. Their roles spanned across a spectrum of healthcare domains, encompassing clinical or bedside nursing, where many began their journeys providing direct patient care within hospitals, clinics, and specialized units. Additionally, some participants carved out niches in specialized care, including roles as nurse anesthetists, nurse practitioners, or nurse educators, requiring advanced training and expertise. Others assumed positions in management and leadership, shouldering responsibilities as nurse managers, charge nurses, or directors of nursing. A significant cohort transitioned into educational roles, contributing to the nurturing of future nurses as instructors.

My nursing career was incredibly diverse. I began as a bedside nurse in a busy urban hospital, where I gained valuable experience in direct patient care. Later on, I specialized as a nurse practitioner, which required advanced training and expertise. My passion eventually led me to become a nurse educator, a role I held until I left the profession. (Participant 6, former nurse, female, left nursing 8 years ago)

However, it is noteworthy that none of the participants have pursued roles in research, policy, or healthcare administration, thus not contributing directly to the shaping of healthcare systems and policies.

We concentrated on caring for patients in hospitals. We didn’t get involved in research, policy, or healthcare administration. Our main role was in direct patient care at the forefront of nursing. (Participant 7, former nurse, male, left nursing 3 years ago).

Moreover, the participants shared a diverse range of memorable experiences from their nursing careers. These included heartwarming patient interactions that highlighted the compassionate side of nursing, instances of medical breakthroughs showcasing clinical expertise, and challenging situations demanding resilience. These narratives illustrated the multifaceted nature of nursing, encompassing both rewarding and demanding aspects of the profession.

I’ll always hold close the memory of the patient whose smile brightened my day, illustrating the immense power of compassion in nursing. (Participant 16, former nurse, male, left nursing 1 year ago).

### Discrepancies between nurses’ expectations and experiences

This analysis provides insight into the discrepancies between the expectations nurses held when entering the profession and the everyday realities they encountered in the field of nursing. A noteworthy point of contrast revolves around the initial aspirations of certain nurses, which were heavily influenced by their strong desire for a balanced lifestyle, financial stability, career growth, and competitive earnings. However, it starkly contrasts with the practical hurdles they confronted, including demanding workloads, limited income, time constraints, and the burden of administrative responsibilities.

Another point of consideration is the variation in motivations related to career stability and financial security. While some nurses may have initially joined the profession with these practical expectations, the pressures, and stresses of the job, such as long hours and demanding patient loads, may have caused disillusionment.

I entered the nursing profession with high hopes of achieving a balanced lifestyle, financial security, and opportunities for career advancement. However, the reality was quite different. The workload was often overwhelming, the pay didn’t meet my expectations. It was a stark contrast to what I had initially envisioned. (Participant 27, former nurse, female, left nursing 3 years ago; participant 4, former nurse, male, left nursing 6 years ago).

When I embarked on my nursing journey, I had little awareness of the distinctions between the roles of a nurse and a medical doctor. Initially, I held the misconception that my role would be similar to that of a medical doctor. (Participant 1, former nurse, male, left nursing 15 years ago).

### Reasons for leaving the nursing profession

The study found that inadequate remuneration, burnout and stress, unfavorable working conditions, job dissatisfaction, strained nurse-doctor relationships, and community misconceptions about nurses’ roles were the main reasons participants left the nursing profession.

#### Inadequate compensation

All the interviewed former nurses revealed that they left the profession due to dissatisfaction with their compensation, given the demanding and emotionally taxing nature of the work, coupled with unequal renumeration.

I departed from the field due to my discontent with the pay and the demanding nature of the job. (Participant 17, former nurse, female, left nursing 4 years ago).

The compensation for the job fell short for me as it couldn’t meet my essential needs. (Participant 32, former nurse, male, left nursing 11 years ago; participant 1, former nurse, male, left nursing 15 years ago; participant 40, former nurse, female, left nursing 5 years ago).

#### Burnout and stress

Burnout and stress are prevalent reasons for nurses leaving. The demanding workloads, long hours, compassion fatigue and emotionally charged situations can lead to physical and emotional exhaustion. Additionally, burnout was exacerbated by the pressure exerted by the work schedules set by the management.

I find it challenging to articulate precisely why I departed, but I distinctly recall grappling with an excessive workload that left me physically and mentally drained. (Participant 10, former nurse, female, left nursing 2 years ago; participant 35, former nurse, male, left nursing 2 years ago).

#### Unfavorable workplace environments

It has come to light that all former nurses who were interviewed stated that both those who departed and those who decided to stay experienced unfavorable working conditions. This encompassed insufficient support, strained interpersonal dynamics, and restricted chances for professional growth and development.

From my own personal experience, I can affirm that both those who left and those who stayed, including myself, encountered an unsatisfactory workplace atmosphere. (Participant 36, former nurse, male, left nursing 1.5 years ago; participant 7, former nurse, male, left nursing 3 years ago).

Insufficient support and strained interactions were widespread among nurses of my time. (Participant 1, former nurse, male, left nursing 3 years ago).

#### Discrepancy between expectations and reality

The majority of the respondents have acknowledged a disparity between their anticipations and the actualities they encountered in practice. This may be due to the absence of career guidance during their academic training and the undervaluation of nurses’ roles within the local community have hastened their decision to leave the profession.

It is true that there was a gap between what I expected and what I experienced in practice. (Participant 33, former nurse, female, left nursing 1 year ago; participant 1, former nurse, male, left nursing 15 years ago; participant 9, former nurse, female, left nursing 2.5 years ago).

My expectations were accurate, but our community held different beliefs. (Participant 35, former nurse, male, left nursing 2 years ago).

#### Strained nurse-doctor collaborations

Strained nurse-doctor collaborations within healthcare settings are a significant concern, affecting both patient care and the overall work environment. The hierarchical nature of healthcare settings was frequently highlighted as a prominent source of tension. Within this hierarchy, nurses often experienced feelings of intimidation or reluctance when it came to expressing concerns or suggestions to physicians, resulting in strained professional relationships. Furthermore, instances of conflicts or disagreements between nurses and doctors were prevalent and, in some cases, persisted without resolution, contributing to ongoing tension within healthcare teams.

In my experience, strained nurse-doctor collaborations have a profound impact on patient care and have been a factor in some nurses leaving the profession. (Participant 7, former nurse, male, left nursing 3 years ago).

I’ve personally faced the challenges of navigating hierarchical tensions in healthcare settings. In fact, this tension was a significant factor in my decision to transition from a nurse to a doctor. (participant 40, former nurse, female, left nursing 5 years ago).

#### Community misconceptions about nurse role

All former nurses have collectively expressed that the community tended to underestimate the expertise and capabilities of nurses, viewing them as mere assistants to doctors rather than highly skilled healthcare professionals.

Throughout my nursing career, I encountered a lack of respect from both the community and fellow healthcare professionals. (Participant 7, former nurse, male, left nursing 3 years ago).

### Impact of nurse attrition on health system

The study elucidated the multifaceted negative impact of nurse attrition on healthcare systems, encompassing factors such as a shortage of nursing staff, diminished quality of patient care, heightened burden on the remaining nurses, extended patient wait time, reduced availability of healthcare services, elevated operational expenses, augmented responsibilities for physicians, and enduring implications for public health.

#### Shortage of nursing staff

Nurse attrition significantly diminishes the magnitude of the nursing profession, and its repercussions extend to the health system itself. A shortage of nursing staff directly affects a health system’s capacity to provide care, straining resources and creating staffing gaps in hospitals and clinics.

I believe that nurse attrition leads to nursing shortages, ultimately jeopardizing the quality of care and the well-being of our patients. (Participant 43, Public hospital director, male, 10 years’ experience; Participant 45, private hospital administrator, male, 10 years’ experience; participant 48, public hospital matron, female, 12 years’ experience).

#### Heightened burden on the remaining nurses

Following nurse attrition, the remaining nurses experience heightened workloads and stress, which can ultimately lead to burnout and reduced job satisfaction. This, in turn, may contribute to increased turnover rates, further exacerbating the nursing shortage within the healthcare system.

I think when some nurses leave, the others have to do a lot more work, which can make them really tired and unhappy. (Participant 43, Public hospital director, male, 10 years’ experience).

I strongly believe that when some nurses quit, it can make the ones left behind have to work much harder and feel stressed. This can make them want to leave their jobs too, making the nursing shortage problem in healthcare even worse. (Participant 44, Private hospital director, male, 3 years’ experience).

#### Extended patient wait time

The study revealed that nurse attrition results in extended waiting times for patients in need of care, potentially causing a backlog of cases, delayed diagnoses, and heightened patient dissatisfaction. This strain on the healthcare system can impede its capacity to deliver timely care.

I strongly feel that when nurses leave, it can make patients wait longer for care and create problems for the healthcare system in providing timely services. (Participant 46, Public-private partners hospital director, male, 4 years’ experience).

#### Diminished quality of patient care

The decline in patient care quality due to nurse attrition has far-reaching consequences for the health system. This includes increased rates of medical errors, patient safety incidents, and a potentially negative reputation for the healthcare facility.

I hold a strong conviction that the diminishing quality of patient care resulting from nurse attrition carries deep and extensive implications for the healthcare system. (Participant 47, Public hospital administrator, male, 7 years’ experience; Participant 43, Public hospital director, male, 10 years’ experience).

#### Reduced availability of healthcare services

The study shed light that the reduced availability of healthcare services due to nurse attrition can result in decreased patient access to critical treatments and services. This can lead to a decline in patient health outcomes and increased healthcare disparities.

I think when nurses are in short supply, it can make it harder for patients to get the treatments they need, which can make people sicker and create more differences in healthcare. (Participant 43, Public hospital director, male, 10 years’ experience).

#### Elevated operational expenses

The need to offer higher salaries and benefits to attract and retain nursing staff can increase the operational expenses of healthcare facilities. This can impact the financial sustainability of the health system and potentially lead to increased costs for patients and payers.

As many nurses left the profession, the remaining ones had higher expectations for compensation. (Participant 43, Public hospital director, male, 10 years’ experience).

During my time as a healthcare administrator, numerous nurses approached me, indicating their intention to leave the profession unless they received improved compensation. (Participant 49, Private hospital director, male, 5 years’ experience).

#### Augmented responsibilities for physicians

As indicated by the study, when nurses exit the profession, physicians must take on additional responsibilities, which can impede their ability to provide specialized care. This situation has the potential to lead to physician burnout and a decline in the overall efficiency of the healthcare system.

In my view, when nurses exit, it puts more on physicians’ plates, affecting their ability to deliver specialized care. (Participant 44, Private hospital director, male, 3 years’ experience).

The departure of nurses places additional strain on the remaining healthcare professionals including doctors & nurses. (Participant 43, Public hospital director, male, 10 years’ experience).

#### Enduring implications for public health

The enduring effects of nurse attrition on public health are significant. It can result in healthcare disparities, decreased access to care, and a diminished ability to respond to public health emergencies or epidemics effectively.

In my perspective, since nurses constitute the largest group among healthcare professionals, their attrition significantly impacts the well-being of the general public. (Participant 50, Private hospital director, male, 13 years’ experience).

## Discussion

The research findings from this qualitative research revealed that the decision to quit nursing is a multifaceted and intricate process that occurs over an extended period. The results suggest that individuals embarking on a nursing career are often motivated by a profound sense of compassion, driving them to positively impact people during their most vulnerable moments, while also holding expectations of equitable remuneration, societal recognition, moral fulfillment, job role flexibility, opportunities for professional growth, and the importance of leisure time. This research aligns with similar studies conducted in various nations [18–22].

Former nurses reflected on their nursing careers, recounting significant experiences in education and patient care practices. Their narratives vividly portrayed the diversity of their professional journey, revealing the multifaceted nature of nursing. These dedicated professionals held roles across various healthcare domains, including clinical or community health nursing. Many of them commenced their careers by providing direct patient care in hospitals, clinics, and specialized units, underscoring the breadth and depth of their experiences in the field. Numerous studies conducted across various nations have consistently uncovered comparable results [23–25]. However, it’s important to highlight that none of the participants have engaged in careers in research, policymaking, or healthcare administration. This may be attributed to the limited access that nurses typically have to policy-making roles and administrative positions in healthcare [26–28].

It also became evident that significant disparities existed between the initial expectations nurses harbored when entering the profession and the actual experiences they encountered in the nursing field. These disparities encompassed various aspects, including monetary compensation, level of respect, workload, opportunities for career advancement, and access to leadership roles. Other studies reported similar findings [29, 30]. This can be explained by the fact that despite nurses serving as the cornerstone of the healthcare system in Somalia, their roles are not accorded the level of recognition and appreciation they rightfully deserve within both the community and the healthcare sector.

In terms of the factors contributing to nurses exiting the nursing profession, the research discovered that inadequate compensation, burnout and stress, unfavorable working conditions, job dissatisfaction, strained nurse-doctor relationships, and a lack of understanding within the community about the nurse’s role continue to be the primary reasons for nurses departing from the profession. Numerous studies carried out across different nations and continents have documented almost similar results [31–36]. These challenges tend to be particularly heightened in countries such as Somalia, which have experienced prolonged periods without effective governance and robust institutional frameworks governing aspects like nursing compensation, lack of equity in workload distribution, poor working conditions, and the strained professional relationships among healthcare cadres.

The study reveals that nurse attrition has several negative impacts on healthcare systems. These include a shortage of nurses, lower quality of patient care, increased workload for the remaining nurses, longer patient wait times, reduced access to healthcare services, higher costs, added responsibilities for doctors, and lasting effects on public health. Consistent findings have been reported across numerous studies conducted in diverse countries [37–40].

These important findings can be attributed to the departure of nurses from their roles, which frequently leads to a shortage of skilled healthcare professionals. This shortage initiates a chain reaction of adverse consequences: a decline in patient care quality, an increased workload for the remaining nurses, extended patient waiting times, and restricted access to healthcare services. Consequently, operational expenses rise, further straining the healthcare system, and placing added responsibilities on physicians. These interconnected challenges have a profound and enduring impact on public health, underscoring the critical role that nurses play within the healthcare ecosystem.

### Strength and limitations

This study demonstrates a notable strength in its comprehensive and nuanced exploration of the factors behind nurse attrition and their implications for healthcare systems in Somalia. Utilizing qualitative research methods, such as conducting in-depth interviews with former nurses and healthcare managers, the study achieved a profound understanding of the multifaceted forces driving nurse attrition. This qualitative approach offered valuable insights into the personal motivations, experiences, and challenges encountered by nurses, shedding light on the systemic consequences of attrition. Nonetheless, the study is not without limitations. Sole reliance on semi-structured interviews as the primary data collection method may limit the breadth of insights. Incorporating additional methods, such as participant observations or focus group discussions, could have provided a more comprehensive understanding of nurse attrition, enriching the study’s findings.

## Conclusion

In conclusion, this qualitative research reveals that nurses’ decisions to leave the profession are complex and multifaceted, shaped by initial aspirations for compassion-driven care and equitable rewards. Former nurses recounted diverse career experiences, but none pursued roles in research or administration. Discrepancies emerged between expectations and realities, including compensation disparities and limited career growth opportunities. Contributing factors to nurse attrition encompassed inadequate monetary compensation, occupational stress and burnout, poor working conditions, job dissatisfaction, strained nurse-doctor relations, and community misunderstandings. These challenges were accentuated in governance-deficient settings like Somalia. Nurse attrition’s negative impacts encompassed staff shortages, reduced care quality, increased workloads, longer patient waits, limited access to healthcare services, higher costs, and burdened physicians, with lasting repercussions on public health, underscoring nurses’ pivotal role in healthcare. To enhance nursing workforce retention, policymakers and healthcare administrators should strategically allocate resources, prioritize comprehensive planning, and specifically target the recognition, support, and retention of nurses within the healthcare framework.

## Supplementary Information

Below is the link to the electronic supplementary material.

Supplementary Material 1

## Data Availability

The data generated and/or analyzed during this study are available from the corresponding author upon reasonable request.
