# Supplementary material for: Unveiling the exodus of nurses in Somalia: exploring the reasons for nurse attrition and the implications for the healthcare system
Source: BMC Nurs. 2026 Jul 14;25:940. doi: 10.1186/s12912-026-05021-z (PMC13647719; doi:10.1186/s12912-026-05021-z)
Supplement: Supplementary file 1 [file 12912_2026_5021_MOESM1_ESM.docx]

**Supplementary file 1**

**DATA COLLECTION TOOL:**

**Exploring the reasons for nurse attrition and the implications for the healthcare system**

I) **Interview guide for Former Nurses**
A. **Introduction and Background:**
1. What is your sex?
2. What is your age (years)
3. Could you share a bit about yourself and your background in nursing?
B. **Expectations of the former nurse**
4. What were your initial expectations and aspirations when you decided to pursue a career in nursing?/ What initially motivated you to pursue a career in nursing?/ Could you elaborate on what drew you to this profession?
C. **Nursing Experience:**
5. Can you walk me through your nursing career, including the different roles you held and the settings you worked in, the type, level & duration?
6. Reflecting on your time as a nurse, could you share some particularly memorable experiences that stand out to you?
7. Do your expectations align with your experiences?

**C. Reasons for Leaving the Nursing Profession**

8. Can you please discuss the factors that ultimately led to your decision to leave the nursing profession?
9. Delving deeper, what were some of your personal motivations for making the decision to leave nursing?
10. Could you share your thoughts on the challenges you faced during your time as a nurse?
II) **Interview guide for Healthcare Managers**

**Introduction and Background**

1. Could you share a bit about yourself, your demographic info (age, gender & qualification) and your current role?

**Nurse Attrition Impact**

3. In your experience, how does nurse attrition impact the healthcare facility that you oversee? What are the noticeable effects on both staff and operations?
4. Could you elaborate on the consequences of nurse turnover specifically in terms of patient care quality and the overall dynamics of your organization?

**Recommendations/Interventions**

5. From your perspective as a healthcare manager, what recommendations do you give to enhance nurse retention and promote job satisfaction among nursing staff?

**Thank you for your time**
